# Youth Mental Health Services Utilization Rates After a Large-Scale Social Media Campaign: Population-Based Interrupted Time-Series Analysis

**DOI:** 10.2196/mental.8808

**Published:** 2018-04-06

**Authors:** Richard G Booth, Britney N Allen, Krista M Bray Jenkyn, Lihua Li, Salimah Z Shariff

**Affiliations:** ^1^ Arthur Labatt Family School of Nursing Western University London, ON Canada; ^2^ Institute for Clinical Evaluative Sciences London, ON Canada

**Keywords:** mental health, youth, adolescent, social media, population health, mass media

## Abstract

**Background:**

Despite the uptake of mass media campaigns, their overall impact remains unclear. Since 2011, a Canadian telecommunications company has operated an annual, large-scale mental health advocacy campaign (Bell Let’s Talk) focused on mental health awareness and stigma reduction. In February 2012, the campaign began to explicitly leverage the social media platform Twitter and incented participation from the public by promising donations of Can $0.05 for each interaction with a campaign-specific username (@Bell_LetsTalk).

**Objective:**

The intent of the study was to examine the impact of this 2012 campaign on youth outpatient mental health services in the province of Ontario, Canada.

**Methods:**

Monthly outpatient mental health visits (primary health care and psychiatric services) were obtained for the Ontario youth aged 10 to 24 years (approximately 5.66 million visits) from January 1, 2006 to December 31, 2015. Interrupted time series, autoregressive integrated moving average modeling was implemented to evaluate the impact of the campaign on rates of monthly outpatient mental health visits. A lagged intervention date of April 1, 2012 was selected to account for the delay required for a patient to schedule and attend a mental health–related physician visit.

**Results:**

The inclusion of Twitter into the 2012 *Bell Let’s Talk* campaign was temporally associated with an increase in outpatient mental health utilization for both males and females. Within primary health care environments, female adolescents aged 10 to 17 years experienced a monthly increase in the mental health visit rate from 10.2/1000 in April 2006 to 14.1/1000 in April 2015 (slope change of 0.094 following campaign, *P*<.001), whereas males of the same age cohort experienced a monthly increase from 9.7/1000 to 9.8/1000 (slope change of 0.052 following campaign, *P*<.001). Outpatient psychiatric services visit rates also increased for both male and female adolescents aged 10 to 17 years post campaign (slope change of 0.005, *P*=.02; slope change of 0.003, *P*=.005, respectively). For young adults aged 18 to 24 years, females who used primary health care experienced the most significant increases in mental health visit rates from 26.5/1000 in April 2006 to 29.2/1000 in April 2015 (slope change of 0.17 following campaign, *P*<.001).

**Conclusions:**

The 2012 *Bell Let’s Talk* campaign was temporally associated with an increase in the rate of mental health visits among Ontarian youth. Furthermore, there appears to be an upward trend of youth mental health utilization in the province of Ontario, especially noticeable in females who accessed primary health care services.

## Introduction

### Background

Over the last decade, the growing use of social media technology has become an important method for many forms of societal communication. Given the broad reach of social media, it has been leveraged as a communication mechanism for a range of different health interventions, including smoking cessation [1], alcohol awareness [2], HIV prevention [3], childhood obesity [4], sexual health practices [5], and mental health awareness [6]. However, it is not certain whether these types of social media campaigns actually influence the behaviors of intended audiences [7,8] or the health care system in measurable ways [9]. Research completed to date provides an incomplete picture regarding the impact of social media used in health campaigns [10,11]. Currently, we know that traditional mass media health campaigns conveyed by television, radio, print advertisements, and outdoor media can generate reasonably effective results at the population level, especially when multiple media interventions are used to target an episodic situation (eg, vaccination) [9,12]. What remains unclear is whether large-scale health awareness campaigns underpinned primarily by social media messaging can also generate measureable behavior change at the population level, especially around sensitive topics such as mental illness and its related stigma.

In Canada, one mental health awareness campaign that has gained significant attention since 2011 is *Bell Let’s Talk* [6,13]. First initiated in February 2011 and led by the Canadian telecommunications company Bell Canada, the *Bell Let’s Talk* campaign has become a yearly event that draws significant volumes of social media traffic related to mental health and stigma awareness. The campaign is hosted by Clara Hughes, a prominent Canadian female athlete who medaled for Canada in both the summer and winter Olympics from 1996 to 2010. By recounting her struggles with depression, the campaign encourages the public to *start a dialogue* to break the silence around mental illness and to support mental health awareness across Canada [14]. To achieve this, the campaign encourages the public to interact with the campaign’s various digital markers via social media on a predetermined day each year. Starting in 2012, the campaign began to leverage social media as a primary mechanism to encourage engagement with the public. During the February 8, 2012 event, the company promised to donate Can $0.05 to mental health research and programing for every retweet of the *@Bell_LetsTalk* campaign tweet (or in later years, any usage of the #BellLetsTalk hashtag on a variety of social media platforms) in addition to any call or SMS text message (short message service, SMS) sent on the Bell Canada network [15] (see Multimedia Appendix 1 for tweet examples from February 8, 2012). The primary goal of the 2012 campaign was to invite Canadians to talk about mental health in an effort to break down stigma that “keeps too many people from seeking the help they need [16].” By the end of the 2012 campaign day, 78,520,284 interactions had been generated, resulting in a donation of Can $3.92 million by Bell Canada to Canadian mental health programs [17]. In more recent years, other events and activities related to the campaign have been held, including a 110 day cross-Canada bike ride by Clara Hughes in 2014 and the expansion of a community fund that provided financial grants to Canadian-registered charities and non-for-profit organizations to support mental health community-based programs and services. The most recent 2017 Bell Let’s Talk campaign event (January 25, 2017) resulted in almost 132 million social media and network interactions (raising Can $6.59 million) [18] and represents what is likely the most widely distributed campaign using elements of social media to broadcast mental health or stigma-reduction messaging in Canada.

Despite the success of *Bell Let’s Talk* and other similar mental health or antistigma campaigns [9,19,20], limited research has focused on evaluating the impact on behavior change. Instead, previous research has focused on evaluating campaign awareness by the public and resulting attitude changes and knowledge uptake [19-23]. Recent evaluation of *Bell Let’s Talk* by Harris/Decima has suggested that the campaign was successful at decreasing stigma and increasing personal awareness in a random telephone survey of Canadian adults (N=1007) over a 5-year period (2011-2015) [24]; however, no examination of the campaign’s influence on behavior change at population level has been undertaken. To our knowledge, population-level behavior change of people in response to a mass media mental health campaign has only been addressed in one study [9], and no known studies exist where social media was used as a primary mechanism of intervention dissemination.

### The Study

Due to the increasing rates of mental illness, especially in youth populations [25-28] who are also heavy users of social media technology [29,30], our population-level analysis sought to evaluate the potential effects of the *Bell Let’s Talk* on adolescent (aged 10-17 years) and young adult populations (aged 10-24 years). We hypothesized that the 2012 *Bell Let’s Talk* campaign would stimulate an increase in mental health service utilization by youth, particularly for females, because of the use of a popular, female athlete role model (ie, Clara Hughes) as the primary campaign spokesperson. Therefore, our primary objectives were to (1) Investigate whether the occurrence of the 2012 *Bell Let’s Talk* campaign was temporally associated with changes in mental health system use by youth aged 10 to 24 years and (2) Examine secular trends in mental health system use by youth over a 10-year period. To our knowledge, social media–enabled mental health awareness campaigns of this size and scale have never been fully examined using population-based methods.

## Methods

### Study Design and Setting

We conducted a cross-sectional time series analysis of all youth aged 10 to 24 years who accessed outpatient mental health services from January 1, 2006 to December 31, 2015 in the province of Ontario, Canada. Residents of Ontario access universal health care, including hospital, diagnostic, and physician services. Research ethics board approval was obtained from Sunnybrook Health Sciences Centre, Toronto. Participant informed consent was not required. This report adheres to the REporting of studies Conducted by using the Observational Routinely-collected health Data statement [31].

### Data Sources

All Ontario residents have access to universally funded health care services and are identifiable through health care administrative records held by the Institute for Clinical Evaluative Sciences (ICES). Within the ICES environment, individual-level health service utilization can be anonymously tracked, linked, and analyzed for research purposes. A unique, encoded identifier permits linkage across several administrative databases. We used the Registered Persons Database (RPDB) to identify all youth aged 10 to 24 years who received outpatient mental health services during the study period. Demographic data and vital status were also obtained from the RPDB. The fee-for-service billings managed by the Ontario Health Insurance Plan (OHIP) were used to identify outpatient mental health visits to (1) Primary health care (family or general practitioners) and (2) Psychiatric services (psychiatrists). Diagnostic codes derived from the International Classification of Diseases, 9th Revision were used to identify all mental health visit diagnoses. Definitions of variables and administrative codes used in this study are detailed in Multimedia Appendices 1-5.

### Study Population

Primary health care mental health and outpatient psychiatric service billings were identified for all youth aged 10 to 24 years from January 1, 2006 to December 31, 2015. Billing records were excluded if they had an invalid provincial health card number; were missing key demographic variables, including age and sex; or were billed to individuals not residing in the province of Ontario. As physicians can bill multiple billing codes for a visit, we collapsed multiple billings by the same physician for the same patient on a single day into one visit. In each month of our 10-year study period (January 1, 2006-December 31, 2015), we identified all youth (adolescents aged 10-17 years and young adults aged 18-24 years, separately) who received outpatient mental health services. Participants were included if they met the age inclusion and had a valid OHIP status. Youth who were missing key demographic variables, including age and sex, or those not residing in the province of Ontario, were excluded from analysis.

### Outcome Measures

Our primary outcomes of interest were the rate of outpatient mental health visits to (1) Primary health care or (2) Psychiatric services, ascertained on a monthly basis and stratified by sex (males and females) and youth groups (adolescents [aged 10-17 years]; young adults [aged 18-24 years]). Eligible outpatient visits for mental health were defined using OHIP billing codes with a mental health diagnosis. Diagnostic codes were selected according to a modified Steele et al [25,32] algorithm for diagnostic, fee, and assessment codes indicative of a mental health system visit (Multimedia Appendices 2 and 3). Secondary outcomes included *new* mental health visits to (1) Primary health care or (2) Psychiatric services. A visit was defined as a *new* visit if the youth did not experience any mental health visit in the previous 12 months. All outcomes are presented as rates, calculated as the total number of visits per month divided by the corresponding youth and sex group population estimates derived from intercensal and postcensal estimates of the Ontario population (Ontario Ministry of Health and Long-Term Care: IntelliHEALTH) [33].

### Population Characteristics

We identified the following patient characteristics: age, sex, neighborhood-based socioeconomic status (neighborhood income quintiles), rurality status based on location of residence, comorbidity status, hospitalizations in the previous year, and diagnosis subgroup for their mental health visit. Income quintiles were based on a neighborhood measure of average household income, determined from their residential postal code and Statistics Canada Census data [34]. Previous hospitalizations were captured from the Discharge Abstract Database, whereas diagnostic subgroups were defined using the diagnosis codes associated with the OHIP databases billings for the mental health visits. Derived diagnostic groupings categorized outpatient diagnoses as either arising from a psychotic disorder, addictions and substance abuse, nonpsychotic disorder, or social problems [25,32] (Multimedia Appendices 2-4). Comorbidity status was defined using the Charlson Comorbidity Index [35]; an index that quantifies comorbidity based on prior hospital admissions. Previous health system use captured the number of outpatient mental health visits in the 12 months before the index mental health visit.

### Statistical Analysis

The study cohort was summarized using descriptive statistics, which included using proportions for categorical data and mean with SD or median with interquartile range for continuous data. Population characteristics were compared at three predefined time points: (1) preintervention, April 2008; (2) peri-intervention, April 2011; and (3) postintervention, April 2014. The three predefined time points were selected a priori to identify any potential demographic variability or nonstable health system utilization patterns of the population. Differences between time periods were assessed using the chi-square test for categorical data and one-way analysis of variance for continuous data. Interrupted time series analysis was performed to examine the impact of the 2012 *Bell Let’s Talk* campaign (February 8, 2012) on each of our primary and secondary outcomes of interest, by sex groups (males and females). The models quantify both a level and slope change following the intervention, while accounting for the autocorrelation of observations. The 2012 campaign was selected as the intervention, as it was the first year that social media was officially leveraged and promoted. The onset date for the intervention was lagged nearly 2 months (until April 1, 2012) to account for the delay that would be required for a patient to schedule and attend a mental health–related physician visit. Analyses were performed at the visit level, and each year was divided into monthly intervals, spanning a total of 120 months during the study period (2006-2015). Autoregressive integrated moving average models were used to adjust for residual autocorrelation. All analyses were performed at the Institute for Clinical Evaluative Sciences using SAS (SAS Institute) statistical software (SAS version 9.3 and SAS EG 6.1) using a Type 1 error rate of 0.05 as the threshold for statistical significance.

### Sensitivity Analysis

A sensitivity analysis was completed using a tracer variable of upper respiratory tract infections (Multimedia Appendix 5), which would not have been influenced by the campaign. This was done to assess whether any observable differences or biases existed within the cohort related to nonmental health system interaction or the presence of billing coding changes in the administrative data record between genders.

## Results

### Participants

In Ontario, the estimated number of youth aged 10 to 24 years during the study time period ranged from 2,576,630 (2006) to 2,564,097 (2015). Two age cohorts were selected for specific examination: (1) adolescents aged 10 to 17 years and (2) young adults aged 18 to 24 years.

#### Adolescents

**Figure 1:**
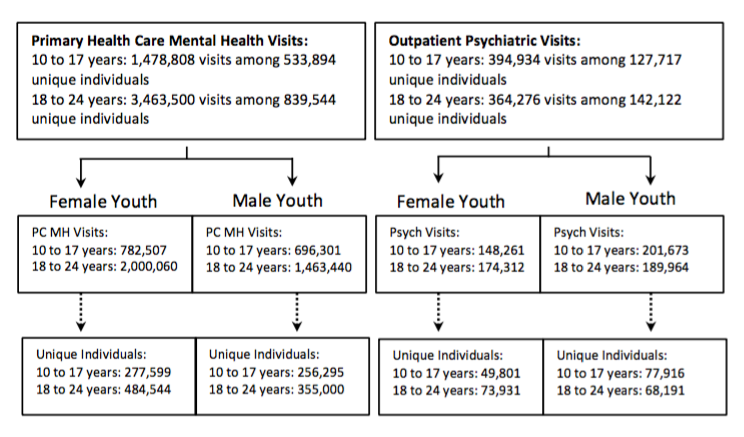
Flowchart detailing mental health visits for primary health care psychiatric services by Ontario youth aged 10 to 17 years and 18 to 24 years from 2006 to 2015. Primary health care mental health and outpatient psychiatric service billings were identified for all youth aged 10 to 24 years from January 1, 2006 to December 31, 2015. Billing records were excluded if they had an invalid provincial health card number; were missing key demographic variables, including age and sex; or were billed to individuals not residing in the province of Ontario. Billings were restricted to one record per patient per day to define a health care visit.

Over the entire study period, a total of 256,295 males and 277,599 females in the age group of 10 and 17 years made visits to primary health care services for mental health reasons, accounting for a total of 696,301 and 782,507 visits for males and females, respectively. Outpatient psychiatric services were accessed over the study period by a total of 77,916 males and 49,801 females, accounting for 201,673 and 148,261 visits, respectively (Figure 1).

#### Young Adults

A total of 355,000 males and 484,544 females in the age group of 18 to 24 years made visits to primary health care services for mental health reasons, accounting for a total of 1,463,440 and 2,000,060 visits for males and females, respectively. Outpatient psychiatric services were accessed over the study period by a total of 68,191 males and 73,931 females, accounting for 189,964 and 174,312 visits, respectively (Figure 1).

### Outcomes

#### Adolescents

**Figure 2:**
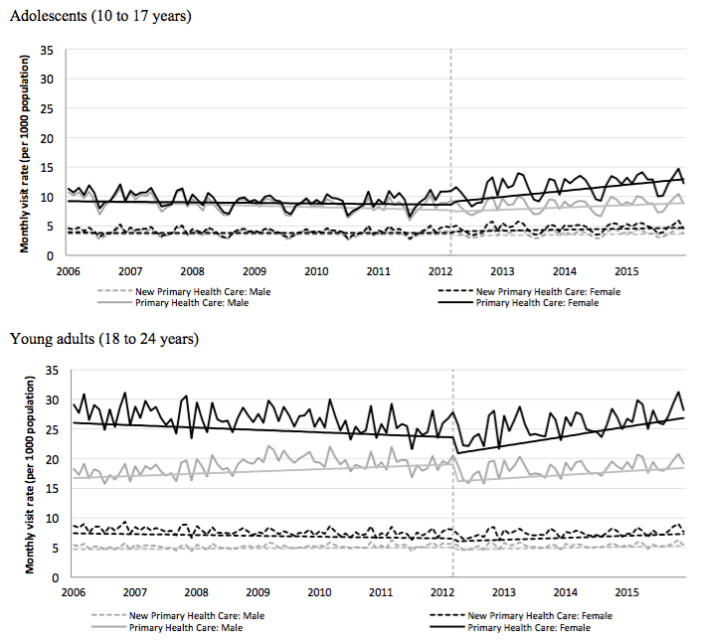
Monthly rate of outpatient mental health visits to primary health care and new mental health visits to primary health care for male and female: (1) adolescents (aged 10-17 years; top panel) and (2) young adults (aged 18-24 years; bottom panel) from 2006 to 2015. Trend lines show slope change after lagged intervention date. Vertical dash line shows lagged intervention date of April 1, 2012.

**Figure 3:**
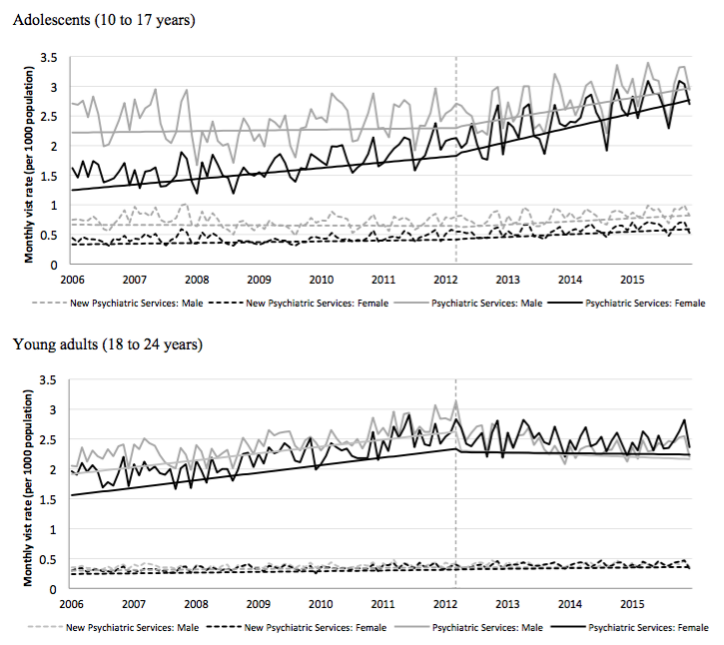
Monthly rate of outpatient mental health visits to psychiatric services and new mental health visits to psychiatric services for male and female: (1) adolescents (aged 10-17 years; top panel) and (2) young adults (aged 18-24 years; bottom panel) from 2006 to 2015. Trend lines show slope change after lagged intervention date. Vertical dash line shows lagged intervention date of April 1, 2012.

The monthly mental health visit rate to primary health care for females increased from 10.2/1000 in April 2006 to 14.1/1000 in April 2015, whereas male adolescents exhibited a moderate change from 9.7/1000 to 9.8/1000 (Figure 2). The monthly mental health visit rate to psychiatric services for female and male youth over the same two time points increased from 1.5/1000 to 3.1/1000 and 2.5/1000 to 3.4/1000, respectively (Figure 3). The monthly rate of new visits to psychiatric services also increased for females (0.42/1000 to 0.71/1000) and males (0.74/1000 to 0.99/1000) over the same time points (Figure 3).

After adjusting for seasonality and correlation between time periods, the campaign was associated with a statistically significant increase in the slope (visit rate) for all four outcomes among both sexes (Figure 2; Table 1). Females exhibited a nearly two-fold change in slope for both mental health visits and *new* mental health visits to primary health care when compared with their male counterparts (females: 0.094, *P*<.001 vs males: 0.052, *P*<.001 and females: 0.02, *P*=.004 vs males: 0.009, *P*=.03, respectively). No statistically significant changes in the levels (magnitude) of the visit rates were observed immediately following the campaign.

#### Young Adults

Among females, the mental visit rate to primary health care increased from 26.5/1000 to 29.2/1000 over the 10-year period. Males also experienced increases from 16.6/1000 to 20.3/1000 over study (Figure 2). The monthly mental health visit rate to psychiatric services for males and females over the same two times points also increased from 2.1/1000 to 2.3/1000 and 1.95/1000 to 2.5/1000 for females and males, respectively (Figure 3). Furthermore, the monthly rate of new visits to psychiatric services increased for females from 0.28/1000 to 0.41/1000 and males from 0.34/1000 to 0.4/1000 (Figure 3).

In young adults, a statistically significant increase in the slope of the primary health care visit rates in females (0.17, *P*<.001), but not males, was observed (Figure 2; Table 1), whereas both exhibited a significant immediate drop in the rates (magnitude change) of primary health care mental health visits following the campaign (males: −2.9/1000 and females: −2.8/1000). This pattern was similar among new primary health care visits. Although the change in slope of new psychiatric services visits remained unchanged for both sexes, both male and female young adults experienced a decrease in the slope, to a plateau, of all psychiatric service visits after April 2012. A significant immediate drop in the rate of psychiatric service visits was observed for males, but not females.

### Tracer

To understand whether our findings may be influenced by other cohort effects, we repeated the analysis using visit rates for upper respiratory tract infections (Multimedia Appendix 5). After adjusting for seasonality and correlation between time periods, no significant differences were observed; either the level or slope changes.

### Descriptive Data

Demographic characteristics for male and female adolescents and young adults who accessed primary health care for mental health purposes are presented in Tables 2 and 3. These demographic characteristics were defined for three a priori-selected time periods: April 2008 (preintervention), April 2011 (peri-intervention), and April 2014 (postintervention).

#### Adolescents

Among adolescents, the mean age of females was higher than that for males for all three time periods; a larger proportion of females were in the age range of 14 to 17 years compared with males (April 2014: 5655/7088, 79.78% vs 3387/5386, 62.88%, respectively). No considerable differences were noted between females and males with regards to the distribution across neighborhood income quintiles. More males had no hospitalizations in the previous 5 years, compared with females. For both males and females, the diagnoses most commonly noted were mood or behavioral complexion (ie, nonpsychotic disorders), followed by social problems, psychotic disorders, and substance use disorders. The median number of mental health visits per month for each of the three monthly intervals was consistently two visits for both males and females (Table 2).

**Table 1:** Interrupted time series parameter estimates for visit rate per 1000 people by visit type (ages 10-17 years; 18-24 years) from January 1, 2006 to December 31, 2015. Visit types were modeled separately for females and males. The campaign level parameter estimate measures the change in the visits from January 1, 2006 to December 31, 2015. The campaign trend estimate measures the change in rate of visit rate between the two time periods.

Visit type			Campaign level estimate (magnitude change)	*P* value	Campaign trend estimate (slope change)	*P* value
**Primary health care visits, age (years)**						
	**10-17**					
		Female	0.47	.30	0.09	<.001
		Male	−0.24	.38	0.05	<.001
	**18-24**					
		Female	−2.80	<.001	0.17	<.001
		Male	−2.90	<.001	0.02	.28
**New primary health care visits, age (years)**						
	**10-17**					
		Female	0.28	.06	0.02	.004
		Male	−0.16	.16	0.01	.03
	**18-24**					
		Female	−0.49	.02	0.04	<.001
		Male	−0.41	.003	0.01	.03
**Psychiatric services, age (years)**						
	**10-17**					
		Female	0.04	.60	0.01	<.001
		Male	0.02	.88	0.01	<.001
	**18-24**					
		Female	−0.05	.40	-0.01	<.001
		Male	−0.32	.002	-0.01	.003
**New psychiatric services, age (years)**						
	**10-17**					
		Female	0.01	.75	.003	.005
		Male	−0.02	.65	0.005	.02
	**18-24**					
		Female	0.005	.64	0.00	.76
		Male	−0.01	.71	0.00	.23

#### Young Adults

For youth in the older cohort, males and females were equally matched in terms of average age. Furthermore, in both males and females, the 22- to 24-year old strata represented the age range where the majority of the mental health–related primary health care interactions occurred (eg, females aged 22-24 years in April 2014 used 48.08%, or 3408 out of 7088 total visits experienced by those aged 18-24 years). No considerable differences were noted between females and males with regards to the distribution across neighborhood income quintiles. For both genders, mood or behavioral disorders (nonpsychotic) were most commonly diagnosed, followed by social, psychotic, and substance use disorders, respectively (Table 3).

**Table 2:** Characteristics of Ontario adolescents aged 10 to 17 years who had a primary health care mental health visit in the months of April 2008, 2011, and 2014, separated by males and females. Population characteristics are presented for three predefined time points: (1) preintervention, April 2008; (2) peri-intervention, April 2011; and (3) postintervention, April 2014. The three predefined time points were used to identify any potential demographic variability or nonstable health system utilization patterns of the population.

Demographics			Male (10-17 years)				Female (10-17 years)			
			Preintervention, April 2008 (N=6132)	Peri-intervention, April 2011 (N=5243)	Postintervention, April 2014 (N=5386)	*P* value	Preintervention, April 2008 (N=6023)	Peri-intervention, April 2011 (N=5439)	Postintervention, April 2014 (N=7088)	*P* value
**Age at index date**						<.001				<.001
	Mean (SD)		13.85 (2.33)	14.18 (2.30)	14.18 (2.34)		14.76 (2.09)	14.87 (2.00)	14.96 (1.92)	
	Median (interquartile range, IQR)		14 (12-16)	15 (12-16)	15 (12-16)		15 (14-16)	15 (14-17)	15 (14-17)	
	**Categories (years), n (%)**					<.001				<.001
		10-11	1315 (21.44)	942 (18.0)	977 (18.1)		668 (11.1)	492 (9.0)	524 (7.4)	
		12-13	1333 (21.74)	969 (18.5)	1022 (18.98)		821 (13.6)	747 (13.7)	909 (12.8)	
		14-15	1566 (25.54)	1398 (26.67)	1339 (24.86)		1785 (29.64)	1655 (30.43)	2247 (31.70)	
		16-17	1918 (31.28)	1934 (36.89)	2048 (38.02)		2749 (45.64)	2545 (46.79)	3408 (48.08)	
**Income-based socioeconomic status, n (%)**						.58				.05
	Quintile 1 (low)		1151 (18.77)	982 (18.7)	988 (18.3)		1115 (18.51)	965 (17.7)	1239 (17.48)	
	Quintile 2		1190 (19.41)	946 (18.0)	955 (17.7)		1178 (19.56)	1052 (19.34)	1302 (18.37)	
	Quintile 3		1219 (19.88)	1065 (20.31)	1110 (20.61)		1194 (19.82)	1117 (20.54)	1381 (19.48)	
	Quintile 4		1275 (20.79)	1140 (21.74)	1150 (21.35)		1231 (20.44)	1166 (21.44)	1555 (21.94)	
	Quintile 5 (high)		1277 (20.83)	1094 (20.87)	1165 (21.63)		1281 (21.27)	1128 (20.74)	1591 (22.45)	
	Missing		20 (0.3)	16 (0.3)	18 (0.3)		24 (0.4)	11 (0.2)	20 (0.3)	
Rural location^a^, n (%)			831 (13.6)	692 (13.2)	724 (13.4)	.84	752 (12.5)	618 (11.4)	929 (13.1)	.23
**Health care system utilization**						<.001				<.001
	**Charlson comorbidity score, n (%)**									
		0	576 (9.4)	520 (9.9)	549 (10.2)		758 (12.6)	707 (13.0)	1132 (15.97)	
		1+	61 (1)	38 (0.7)	49 (0.9)		66 (1)	54 (1)	64 (0.9)	
	No hospitalizations, n (%)		5495 (89.61)	4685 (89.36)	4789 (88.92)		5199 (86.32)	4678 (86.01)	5892 (83.13)	
Previous mental health visit in the past 12 months, n (%)			3119 (50.86)	2651 (50.56)	2774 (51.50)	.61	2924 (48.55)	2747 (50.51)	4010 (56.57)	<.001
	**Number of mental health visits during monthly interval **					.05				<.001
		Mean (SD)	2.31 (0.88)	2.24 (0.60)	2.19 (0.53)		2.52 (1.33)	2.28 (0.71)	2.21 (0.51)	
		Median (IQR)	2 (2-2)	2 (2-2)	2 (2-2)		2 (2-2)	2 (2-2)	2 (2-2)	
**Diagnosis group**						<.001				<.001
	**Diagnosis groups, n (%)**								
		Psychotic	168 (2.7)	160 (3.1)	176 (3.3)		101 (1.7)	98 (2)	148 (2.1)	
		Substance	150 (2.4)	105 (2.0)	69 (1)		91 (2)	77 (1)	51 (0.7)	
		Nonpsychotic	5420 (88.39)	4711 (89.85)	4907 (91.11)		5557 (92.26)	5050 (92.85)	6713 (94.71)	
		Social	394 (6.4)	267 (5.1)	234 (4.3)		274 (4.5)	214 (3.9)	176 (2.5)	

^a^Rural location defined as residential areas outside the commuting zone of a city with a population ≥10,000. Missing data on fewer than 0.1% per year.

**Table 3:** Characteristics of Ontario young adults aged 18 to 24 years who had a primary health care mental health visit in the months of April 2008, 2011, and 2014, separated by males and females. Population characteristics are presented for three predefined time points: (1) preintervention, April 2008, (2) peri-intervention, April 2011, and (3) postintervention, April 2014. The three predefined time points were used to identify any potential demographic variability or nonstable health system utilization patterns of the population.

Demographics			Male (18-24 years)				Female (18-24 years)			
			Preintervention, April 2008 (N=9226)	Peri-intervention, April 2011 (N=9492)	Postintervention, April 2014 (N=10,810)	*P* value	Preintervention, April 2008 (N=13,946)	Peri-intervention, April 2011 (N=12,818)	Postintervention, April 2014 (N=14,830)	*P* value
**Age at index date**						<.001				<.001
	Mean (SD)		21.23 (1.96)	21.12 (1.96)	21.10 (1.97)		21.22 (1.96)	21.13 (1.97)	21.03 (1.96)	
	Median (interquartile range, IQR)		21 (20-23)	21 (19-23)	21 (19-23)		21 (20-23)	21 (19-23)	21 (19-23)	
	**Categories (years), n (%)**					<.001				<.001
		18-19	2197 (23.81)	2383 (25.11)	2870 (26.55)		3357 (24.07)	3285 (25.63)	4014 (27.07)	
		20-21	2635 (28.56)	2896 (30.51)	3118 (28.84)		3996 (28.65)	3733 (29.12)	4428 (29.86)	
		22-24	4394 (47.63)	4213 (44.38)	4822 (44.61)		6593 (47.28)	5800 (45.25)	6388 (43.07)	
**Income-based socioeconomic status, n (%)**						<.001				<.001
	Quintile 1 (low)		2094 (22.70)	2058 (21.68)	2198 (20.33)		3250 (23.30)	2915 (22.74)	3171 (21.38)	
	Quintile 2		1946 (21.09)	1786 (18.82)	2029 (18.77)		2836 (20.33)	2525 (19.70)	2748 (18.53)	
	Quintile 3		1674 (18.14)	1765 (18.59)	2035 (18.83)		2603 (18.66)	2315 (18.06)	2866 (19.33)	
	Quintile 4		1714 (18.58)	1826 (19.24)	2208 (20.43)		2488 (17.84)	2506 (19.55)	2968 (20.01)	
	Quintile 5 (high)		1756 (19.03)	2010 (21.18)	2300 (21.28)		2711 (19.44)	2503 (19.53)	3014 (20.32)	
	Missing		42 (0.5)	47 (0.5)	40 (0.4)		58 (0.4)	54 (0.4)	63 (0.4)	
Rural location^a^, n (%)			881 (9.5)	867 (9.1)	993 (9.2)	.39	1335 (9.57)	1118 (8.72)	1342 (9.05)	.13
**Health care system utilization**						<.001				<.001
	**Charlson comorbidity score, n (%)**																	
		0	1295 (14.04)	1094 (11.53)	1158 (10.71)		3525 (25.28)	2857 (22.29)	2970 (20.03)	
		1+	99 (1)	92 (1)	94 (0.9)		162 (1.1)	156 (1.3)	196 (1.3)	
	No hospitalizations, n (%)		7832 (84.89)	8306 (87.51)	9558 (88.42)		10258 (73.56)	9805 (76.49)	11664 (78.65)	
Previous mental health visit in the past 12 months, n (%)			5634 (61.03)	6023 (63.45)	7084 (65.53)	<.001	8793 (63.05)	8289 (64.67)	9856 (66.46)	<.001
	**Number of mental health visits during monthly interval **					<.001				<.001
		Mean (SD)	3.04 (1.68)	2.92 (1.58)	2.50 (1.09)		2.71 (1.35)	2.63 (1.22)	2.39 (0.86)	
		Median (IQR)	2 (2-4)	2 (2-3)	2 (2-3)		2 (2-3)	2 (2-3)	2 (2-2)	
**Diagnosis group**						<.001				<.001
	**Diagnosis groups, n (%)**									
		Psychotic	497 (5.4)	509 (5.4)	658 (6.1)		341 (2.4)	350 (2.7)	447 (3.0)	
		Substance	1342 (14.55)	1156 (12.18)	791 (7.3)		960 (6.9)	795 (6.2)	548 (3.7)	
		Nonpsychotic	7152 (77.52)	7579 (79.85)	9112 (84.29)		12235 (87.73)	11354 (88.58)	13509 (91.09)	
		Social	235 (2.5)	248 (2.6)	249 (2.3)		410 (2.9)	319 (2.5)	326 (2.2)	

^a^Rural location defined as residential areas outside the commuting zone of a city with a population ≥10,000. Missing data on fewer than 0.1% per year.

## Discussion

### Principal Findings

From 2006 to 2015, the rates of outpatient mental health use by youth aged 10 to 24 years in the province of Ontario increased for both males and females. The 2012 *Bell Let’s Talk* was temporarily associated with increases in the trends of outpatient mental health visits, especially within the adolescent female cohort. Although no discernable difference in the immediate change in the rate of mental health visits was observed among the adolescent groups, young adults exhibited a slight drop in most outpatient mental health visits, followed by a moderate increase or plateauing of rates.

### Comparison With Prior Work

Although we were unable to directly measure aspects related to the public’s awareness of the *Bell Let’s Talk* campaign or their reactions to its messaging in this study, indirect evidence of the campaign’s penetration was captured by a 2015 Harris/Decima study [24]. A majority of participants in this random telephone survey of Canadian adults (N=1007) reported that they believed the campaign was successful at decreasing stigma (57% agreement, 574/1007), increasing personal awareness (81% agreement, 816/1007), and positively changed attitudes toward mental health (70% agreement, 704/1007) over a 5-year period [24]. Although there appears to preliminary evidence of the campaign’s penetration, immediate reaction to the campaign was not observed within health care administrative data. As the *Bell Let’s Talk* campaign was primarily designed to generate awareness surrounding mental health and stigma, the lack of a substantive step change in health care utilization from normal levels is not surprising. Other research exploring the effects of mental health campaigns using social media discovered that changes related to campaign awareness, perceptions of stigma, and behavior intention toward future mental health–related situations were the only measurable variables reactive to the Web-based messaging [36-39]. As the findings of this study demonstrated trend increases related to outpatient mental health visits (slope changes pre- and postintervention), this suggests that the 2012 *Bell Let’s Talk* campaign generated awareness related to a gradual change in behavior, rather than immediately triggering individuals with latent mental health concerns to seek formal mental health services. Therefore, although we hypothesized that the campaign would encourage individuals to seek mental health services, the real outcomes of this campaign were likely more related to societal awareness, rather than discrete outpatient mental health system utilization.

Consistent with other research, there appears to be an upward trend toward mental health services use in youth populations in Ontario [25,28,40], especially noticeable in females who accessed primary health care services. Previous research exploring gender differences related to youth accessing mental health have identified females as possessing greater willingness to seek help [41]. This alone does not provide sufficient explanation related to the further increased mental health visit rate trend observed in females compared with males. Given the growing public literacy related to mental illness [42] and the increasing use of social media for all forms of communication (including discussion related mental health) [20,43], a range of extraneous variables not specifically measured in this study may offer explanatory insights related to the observed increasing mental health utilization slopes. One such promising extraneous factor was the increasing diversity of social media technology that emerged during the latter years of the study. Previous research has demonstrated that usage of certain social media technologies by youth populations can significantly influence body image conception and psychological or mental health well-being [44-48]. With the increasing variety of social media platforms available over the last 5 years (ie, Instagram, Vine, Snapchat, Tinder, and Grindr), further exploration of these forms of technology and their potential influence on youth mental health well-being should be examined.

Currently, we are unaware of any other studies that have utilized population-level data to examine the impact of a mental awareness and stigma campaign propagated largely through social media. Cheng and colleagues’ [9] examination of the impact of a 2-month long, mass media mental health campaign (ie, *Transforming Lives*) on psychiatric emergency department utilization serves as the closest comparator with this study. Although their study focused on psychiatric emergency department visits associated with a mass media mental health campaign, differences in the structure and underpinning of the respective campaigns should be noted. The 2-month mass media campaign (ie, radio, website, newspaper, and Toronto public transportation advertising) examined by Cheng and colleagues [9] was executed by a Toronto-based (Ontario, Canada) hospital organization and did not possess an explicit promise of financial donation for interaction with the campaign; the day-long 2012 *Bell Let’s Talk* campaign was operated nationally by a large Canadian telecommunication company and encouraged participation through Can $0.05 donations for interactions with a campaign-specific social media metadata tag. These salient differences between the respective complexion and scale of campaigns should be kept in mind when comparing findings between the two interventions. Furthermore, although utilizing similar time series methods, Cheng and colleagues’ [9] study was more specific in sampling than this current examination of the *Bell Let’s Talk* campaign. Cheng and colleagues [9] examined visit rates at seven hospitals in the Greater Toronto area (Ontario, Canada) and reported that the *Transforming Lives* mass media campaign was significantly associated with an increased trend in the utilization of the psychiatric emergency department. Our study complements Cheng and colleagues’ [9] findings, suggesting that mass media campaigns such as *Bell Let’s Talk* can have potential influential effects on the use of mental health services at population levels. Further refinement will need to be undertaken to determine whether specific youth cohorts within the province of Ontario were influenced differently by the *Bell Let’s Talk* campaign and how the growing presence of antistigma and mental health awareness in society may be influencing rates of mental health system utilization.

### Limitations

With any ecological study, there are several limitations that need to be explicitly delineated. First, we assessed all outpatient mental health use in this study using administrative data. Due to the nature of the data, it was impossible to measure illness severity for individual interactions with the health system. This limitation prevented deeper analysis of youths’ individual presentations and their usage of outpatient mental health services. Second, emergency department utilization for mental health services was not included in this study. Although emergency departments are an important point of contact for mental health services, only outpatient mental health visits were examined in this study to focus on planned or scheduled interactions that would be better representative of purposeful behavior change in reaction to the *Bell Let’s Talk* campaign messaging. Third, given the broad inclusion criteria used in cohort development (ie, all youth eligible for universal health care), it was not possible to determine the influence of the 2012 *Bell Let’s Talk* campaign on specific population subsets. Further research using smaller, specific, homogenous population cohorts (ie, depression and eating disorders) will be important to determine if there are any epidemiological signals that more wholesomely associate with the campaign. Finally, although the 2012 *Bell Let’s Talk* campaign was associated with an increase in the trends of outpatient mental health visits, other population, environment, and societal extraneous variables may have also influenced the observed changes. Furthermore, given the growing size, scale, and social media embeddedness of the *Bell Let’s Talk* campaign each subsequent year since 2012 [24], the potential cumulative influence of the campaign and its related events and activities (ie, cross-Canada bike ride event; Community Fund) on people over time was not considered in this analysis and should be explored in a future examination.

### Conclusions

This study is one of the first population-level analyses of mental health utilization associated with a large-scale social media campaign targeting mental health awareness and antistigma. The use of health care administrative data allowed examination of mental health system utilization by those aged 10 to 24 years in the province of Ontario over a decade long period. Due to this uniqueness, the study provides important methodological implications for researchers wishing to ascertain the efficacy of social media and other digitally enabled media campaigns operated at population level. With the growing recognition of the communicative power of social media, further research is required to generate more precise modeling techniques to better understand their effects on population and public health [49]. Furthermore, through subgroup analyses sensitive to gender and age, we uncovered that trends in youth mental health utilization (especially for both female age cohorts who used primary health care for mental health services) have significantly increased since 2012. We are unclear of the mechanism related to this sharp trend increase for females, but feel this emergent finding requires immediate focus. Given recent emphasis on gender and sex within health sciences research [50,51], exploration of the relation to mental health system utilization is important for both practice and policy.

## Appendix

Multimedia Appendix 1 Tweet examples from February 8, 2012, including the initation Bell Let’s Talk tweet (top tweet) prompting Twitter users to retweet elements of the @Bell_LetsTalk message and account to generate 5 cent dontations.

Multimedia Appendix 2 Selected OHIP fee codes for assessments, consultations, and clinical specialities that indicate possible mental health system interaction. OHIP: Ontario Health Insurance Plan.

Multimedia Appendix 3 Selected ICD-9 codes in the OHIP database that indicate possible mental health system interaction. Modified from the Steele et al algorithm. OHIP: Ontario Health Insurance Plan.

Multimedia Appendix 4 Source and description of patient variables.

Multimedia Appendix 5 Upper respiratory infection (URI) tracer, to assess for any observable differences or biases within the cohort related to nonmental health system interaction or the presence of billing coding changes in the administrative data record between genders.

## Abbreviations

ICES: Institute for Clinical Evaluative Sciences
OHIP: Ontario Health Insurance Plan
RPDB: Registered Persons Database
