# Supplementary material for: Youth Mental Health Services Utilization Rates After a Large-Scale Social Media Campaign: Population-Based Interrupted Time-Series Analysis
Source: JMIR Ment Health. 2018 Apr 6;5(2):e27. doi: 10.2196/mental.8808 (PMC5938692; doi:10.2196/mental.8808)

Appendix 1. Tweet examples from February 8, 2012, including the initation Bell Let's Talk tweet (top tweet) prompting Twitter users to retweet elements of the @Bell_LetsTalk message and account to generate 5 cent dontations.

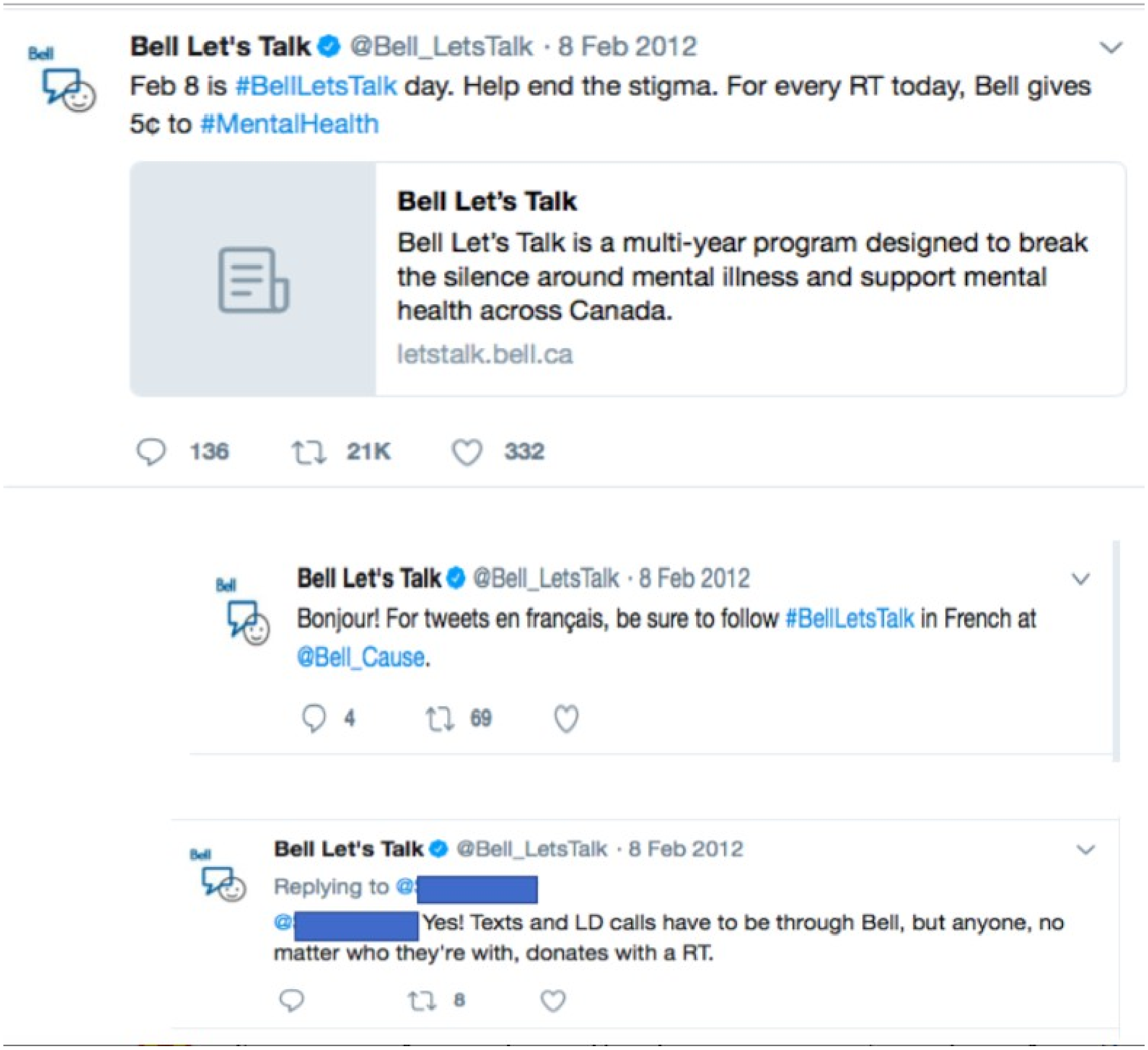

Supplement: Multimedia Appendix 1 [file mental_v5i2e27_app1.pdf]
