# Supplementary material for: Youth Mental Health Services Utilization Rates After a Large-Scale Social Media Campaign: Population-Based Interrupted Time-Series Analysis
Source: JMIR Ment Health. 2018 Apr 6;5(2):e27. doi: 10.2196/mental.8808 (PMC5938692; doi:10.2196/mental.8808)
Supplement: Multimedia Appendix 2 [file mental_v5i2e27_app2.pdf]

Appendix 2. Selected OHIP Fee Codes for Assessments, Consultations, and Clinical Specialities that Indicate Possible Mental Health System Interaction.

OHIP Code	Type of Assessment, Consultation or Practitioner Specialty	Description
K005	Primary mental healthcare - Individual care (30 mins)	General assessment and consultations
K007	Psychotherapy	
K623	Form 1 (Application for Psychiatric Assessment)	
A001	Minor assessment	
A003	General assessment	
A004	General re-assessment	
A005	Consultation	
A006	Repeat consultation	
A007	Intermediate assessment	
A905	Limited consultation	
A190	Special psychiatric consultation	Psychiatric services specific consultations
A193	Specific assessment (psych)	
A194	Partial assessment (psych)	
A195	Consultation (psych)	
A196	Repeat consultation (psych)	
A395	Limited consultation (psych)	
A260	Special paediatric consultaton	Paediatric psychiatric services specific consultations
A265	Paeds consultation	
A662	Extended special paediatric consultation	
K122	Paeds individual developmental and/or behavioural care	
K123	Paeds family developmental and/or behavioural care	
00	Family practice and general practice	Practitioner clinical specialties
05	Community medicine	
26	Paediatrics	
19	Psychiatry	
