# Supplementary material for: Youth Mental Health Services Utilization Rates After a Large-Scale Social Media Campaign: Population-Based Interrupted Time-Series Analysis
Source: JMIR Ment Health. 2018 Apr 6;5(2):e27. doi: 10.2196/mental.8808 (PMC5938692; doi:10.2196/mental.8808)
Supplement: Multimedia Appendix 3 [file mental_v5i2e27_app3.pdf]

Appendix 3. Selected ICD-9 Codes in the OHIP Database that Indicate Possible Mental Health System Interaction. Modified from the Steele et al Algorithm.

OHIP Diagnostic Codes (ICD-9)	Code Description	Type of disorder or problem
295	Schizophrenia	Psychotic disorders
296	Manic depressive psychosis, involutional melancholia	
297	Paranoid states	
298	Other psychoses	
299	Childhood psychoses (e.g., autism)	
291	Alcoholic psychosis, delirium tremens, Korsakov's psychosis	Substance use disorders
292	Drug psychosis	
303	Alcoholism	
304	Drug dependence, drug addiction	
305	Tobacco abuse	
300	Anxiety neurosis, hysteria, neurasthenia, obsessive compulsive neurosis, reactive depression	Non-psychotic disorders
301	Personality disorders (e.g., paranoid personality, schizoid personality, obsessive compulsive personality)	
306	Psychosomatic disturbances	
307	Habit spasms, tics, stuttering, tension headaches, anorexia nervosa, sleep disorders, enuresis	
309	Adjustment reaction	
311	Depressive or other non-psychotic disorders, not elsewhere classified	
313	Behaviour disorders of childhood and adolescence	
314	Hyperkinetic syndrome of childhood	
897	Economic problems	Social problems
898	Marital issues	
899	Parent-child issues	
900	Problems with aged parents or in-laws	
901	Family disruption/divorce	
902	Education problems	
904	Social maladjustment	
905	Occupational problems	
906	Legal problems	
909	Other problems of social adjustment	
