# Supplementary material for: Youth Mental Health Services Utilization Rates After a Large-Scale Social Media Campaign: Population-Based Interrupted Time-Series Analysis
Source: JMIR Ment Health. 2018 Apr 6;5(2):e27. doi: 10.2196/mental.8808 (PMC5938692; doi:10.2196/mental.8808)
Supplement: Multimedia Appendix 4 [file mental_v5i2e27_app4.pdf]

Appendix 4: Source and Description of Patient Variables.

Variable	Source	Classification or Description
Age	RPDB	Strat: from 10-11;12-13;14-15;16-17;18-19;20-21;22-24
Sex	RPDB	Binary: male or female
Income quintile	RPDB	Quintiles of neighborhood-level income (1 = lowest income to 5 = highest income)
Rurality	RPDB	Rurality based on residence location postal code
Charlson Comorbidity Index	DAD	Comorbidity index derived from prior hospital admissions, look back five years
Number of mental health visits within the previous year	DAD<br>OHIP<br>OMHRS	Look back 12 months for any previous mental health visit
Visit diagnosis subgroup	OHIP	Modified Steele et al [25,32] diagnostic groupings of psychotic disorder, substance use disorders, non-psychotic disorders, social problems
