# Supplementary material for: Youth Mental Health Services Utilization Rates After a Large-Scale Social Media Campaign: Population-Based Interrupted Time-Series Analysis
Source: JMIR Ment Health. 2018 Apr 6;5(2):e27. doi: 10.2196/mental.8808 (PMC5938692; doi:10.2196/mental.8808)

Appendix 5. Upper Respiratory Infection (URI) Tracer, to assess for any observable differences or biases within the cohort related to non-mental health system interaction or the presence of billing coding changes in the administrative data record between genders.

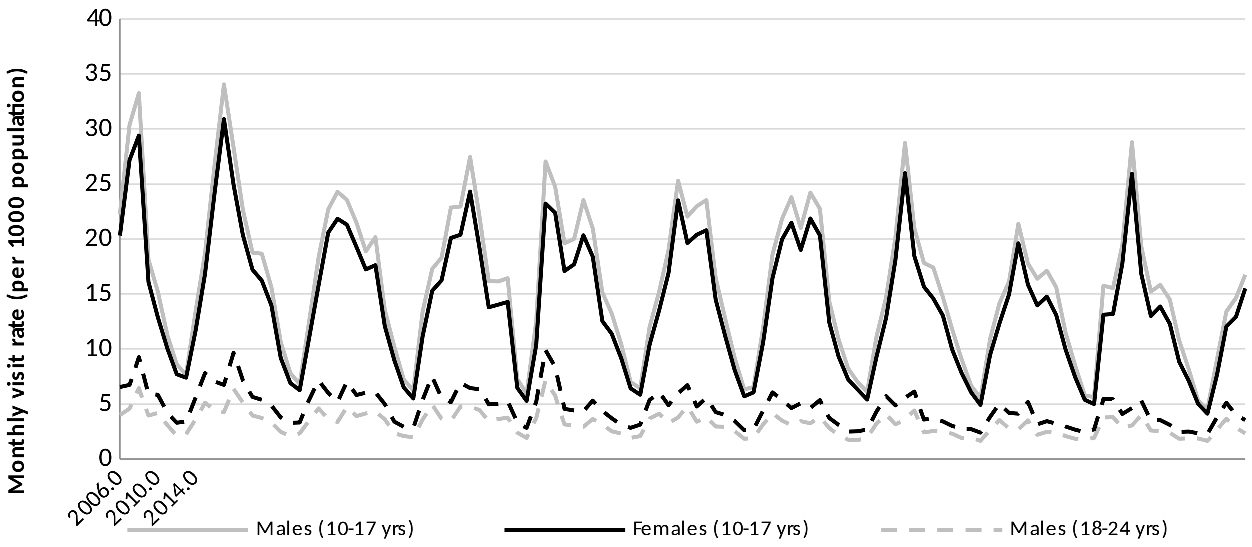

Supplement: Multimedia Appendix 5 [file mental_v5i2e27_app5.pdf]
